# Epidemiological patterns and trends of respiratory syncytial virus in Guangdong Province, China, 2019–2023

**DOI:** 10.3389/fpubh.2026.1851186

**Published:** 2026-07-31

**Authors:** Jinbin Chen, Haoran Qiu, Minfei Yu, Juncong Xiao, Wenxiang Jin, Yuanyin Huang, Bingyan Niu, Dongni Lin, Yichang Weng, Yi Wen, Zhiqi Zeng, Zifeng Yang, Xiaoyan Deng

**Affiliations:** 1 KingMed School of Laboratory Medicine, Guangzhou Medical University, Guangzhou, Guangdong, China; 2 Engineering Technology Research Center of Intelligent Diagnosis for Infectious Diseases in Guangdong Province, Guangzhou, Guangdong, China; 3 Guangzhou Key Laboratory for Clinical Rapid Diagnosis and Early Warning of Infectious Diseases, Guangzhou, Guangdong, China; 4 China-Portugal Artificial Intelligence and Public Health Technologies Joint Laboratory, Guangzhou Medical University, Guangzhou, Guangdong, China; 5 Guangzhou National Laboratory, Guangzhou, Guangdong, China; 6 State Key Laboratory of Respiratory Disease, National Clinical Research Center for Respiratory Disease, The First Affiliated Hospital of Guangzhou Medical University, Guangzhou Medical University, Guangzhou, Guangdong, China; 7 Kingmed Diagnostics, Guangzhou, Guangdong, China

**Keywords:** distributed lag non-linear model, respiratory syncytial virus, RSV transmission, SHAP, XGBoost

## Abstract

**Background:**

Respiratory syncytial virus (RSV) is a major cause of acute respiratory tract infections worldwide. The COVID-19 pandemic and associated non-pharmaceutical interventions (NPIs) substantially altered the transmission dynamics of respiratory viruses. However, post-pandemic changes in RSV epidemiology and their associations with meteorological factors remain insufficiently characterized in subtropical regions of China.

**Method:**

Based on 38,541 RSV test records collected in Guangdong Province from July 1, 2019 to July 2, 2023, the epidemic characteristics were analyzed across the pre-pandemic, pandemic, and post-pandemic phases. The shortest main epidemic period in each season was identified using a sliding window approach. Monthly percentage change was used to quantify temporal variations across epidemic seasons. Meteorological data were integrated to assess temporal trends and correlations. A Distributed Lag Non-linear Model (DLNM) was applied to quantify non-linear exposure–response relationships and lag effects of meteorological factors. An XGBoost model combined with SHAP was used for feature attribution and performance evaluation.

**Results:**

RSV positivity rates exhibited pronounced seasonal and interannual variability throughout the study period. Epidemic periods ranged from 7 to 10 months and frequently extended beyond the traditional winter–spring season. Overall positivity rates increased from 8.19% in the pre-pandemic phase to 13.84% during the pandemic phase and 19.33% in the post-pandemic phase. Significant differences were primarily observed among children younger than 6 years. Multiple meteorological variables were associated with RSV activity. DLNM analyses identified non-linear threshold effects and short-term lag effects (0–3 weeks), with elevated RSV risk associated with specific ranges of temperature, dew point temperature, and precipitation. XGBoost models captured major temporal patterns in RSV activity, with lagged RSV indicators contributing most strongly to predictive performance, whereas meteorological variables provided modest incremental predictive value.

**Conclusion:**

RSV activity in Guangdong during 2019–2023 was characterized by prolonged epidemic periods and altered seasonal patterns following the COVID-19 pandemic. Meteorological factors demonstrated non-linear and lagged associations with RSV activity, highlighting their potential role in shaping transmission dynamics. The combined DLNM and XGBoost-SHAP framework provided complementary insights into climate-related risk patterns and temporal forecasting of RSV activity. Continued surveillance and external validation are needed to determine whether the observed seasonal changes represent a stable long-term pattern and to improve the generalizability of predictive models.

## 1 Introduction

Respiratory Syncytial Virus (RSV) is a major etiological agent of acute respiratory tract infections (ARTIs) across all age groups and imposes a substantial global health and economic burden (1, 2). It is estimated that approximately 33 million children under five years of age are infected with RSV annually, among whom 3–4 million require hospitalization and nearly 100,000 die each year, with the overall disease burden continuing to increase (3, 4). Previous studies have demonstrated marked seasonality in RSV activity (5–7): In temperate regions, epidemics typically peak in winter, whereas in subtropical regions transmission generally begins in October–November, reaches a peak in January–February of the following year, and persists through April–May (8, 9). Surveillance data from multiple regions in China have similarly documented a predominance of winter–spring epidemics (10, 11). The COVID-19 pandemic and the implementation of non-pharmaceutical interventions (NPIs) have substantially altered the transmission dynamics of respiratory pathogens (12). While suppressing SARS-CoV-2 circulation, reduced natural exposure to common respiratory viruses such as RSV has led to an ‘immunity gap’, with several countries reporting seasonal shifts and atypical outbreaks of RSV (13). In Beijing, China, extended epidemic periods and off-season resurgence have also been observed (14). These findings suggest that RSV seasonality in temperate regions has been reconfigured; however, its evolutionary trajectory in subtropical monsoon climates, such as Guangdong Province, remains insufficiently characterized. Given that meteorological factors are recognized as key environmental drivers of RSV seasonality (15), phase-specific differences during the pandemic and regional climatic conditions—characterized by high humidity, abundant rainfall, and complex weather patterns—may jointly influence transmission dynamics. Whether epidemic duration has prolonged and whether the traditional winter–spring peak has shifted in this setting warrant systematic evaluation.

In the post-COVID-19 context, the activity of RSV demonstrates non-linear and non-stationary patterns. This characteristic restricts the ability of conventional linear models to accurately capture the complex exposure–lag relationships and attain high predictive accuracy. Therefore, it is crucial to quantify the potential non-linear threshold effects and short—term lag effects of meteorological factors. Simultaneously, it is necessary to develop analytical frameworks that combine predictive performance with interpretability. Based on the RSV surveillance data in Guangdong Province from 2019 to 2023, which covers the pre-pandemic, pandemic, and post-pandemic phases, this study aims to: (1) characterize the temporal evolution of RSV epidemiological patterns; (2) quantify the non-linear lag associations between key meteorological factors and RSV activity; and (3) develop an interpretable predictive model to support regional RSV surveillance and early warning strategies.

## 2 Materials and methods

### 2.1 RSV data sources

RSV laboratory testing records were retrospectively collected from KingMed Diagnostics (KMD) Laboratory in Guangzhou, one of the largest independent clinical laboratory networks in China, covering the period from July 2019 to June 2023. A total of 38,541 individuals who underwent RSV testing were included. The participating institutions included primary, secondary, and tertiary hospitals distributed across multiple prefecture-level cities throughout Guangdong Province. Specimens were referred from a broad range of healthcare settings and were not restricted to a single hospital, city, or surveillance site. The geographic distribution of submitted specimens generally corresponded to the population distribution and healthcare utilization patterns across Guangdong Province (Supplementary Table S1). Eligibility criteria comprised fever (≥ 37.3 °C) accompanied by at least one respiratory symptom, such as cough, sore throat, rhinitis, or dyspnea. All patients meeting the inclusion criteria and undergoing physician-ordered laboratory testing were included. De-identified retrospective data were obtained, including sample ID, age, sex, testing date, and test result (positive/negative). RSV detection was performed using reverse transcription polymerase chain reaction (RT-PCR) in accordance with standardized operating procedures. The study protocol was approved by the KingMed Medical Ethics Committee (ID: 2024220).

### 2.2 Meteorological data

Meteorological data were obtained from the National Oceanic and Atmospheric Administration (NOAA, https://www.noaa.gov/). Data from all meteorological monitoring stations within Guangdong Province were extracted, and average values for each indicator were calculated for analysis. The meteorological variables included mean temperature (TEMP, °C), mean temperature difference (TD, °C), dew point temperature (DEWP, °C), mean sea-level pressure (SLP, hPa), mean visibility (VISIB, km), mean wind speed (WDSP, m/s), maximum sustained wind speed (MXSPD, m/s), and precipitation (PRCP, mm). Detailed methodological procedures are provided in the Supplementary Tables S2–S4.

### 2.3 Definition of study periods and RSV seasonal characteristics

#### 2.3.1 COVID-19 phases definition

To evaluate the impact of COVID-19 on RSV epidemiological patterns, the study period was divided into three stages based on previous literature (16):

1. COVID-19 Pre-pandemic Stage (Stage I): July 1, 2019—January 26, 2020 (2019-W27 ~ 2020 W4)
2. COVID-19 Pandemic Stage (Stage II): January 27, 2020—January 8, 2023 (2020-W5 ~ 2023-W1), period of strict non-pharmaceutical interventions.
3. COVID-19 Post-pandemic Stage (Stage III): January 9, 2023—July 2, 2023 (2023-W2 ~ 2023-W26), following adjustment of control measures.

The phase boundaries were defined according to major national COVID-19 policy transitions in China. January 26, 2020 was selected as the start of the pandemic stage, corresponding to the implementation of nationwide non-pharmaceutical interventions (NPIs), whereas January 8, 2023 was defined as the beginning of the post-pandemic stage, corresponding to the adjustment of COVID-19 prevention and control policies and the relaxation of major NPIs. In this study, the term “post-pandemic stage” refers to the period following the relaxation of major COVID-19 control measures and does not imply the complete cessation of SARS-CoV-2 transmission.

#### 2.3.2 RSV epidemic season definition

The peak epidemic period within each epidemic season was defined as the shortest consecutive time interval during which the cumulative positivity rate accounted for 80% of the total positive cases in that season. RSV epidemic season was defined as July to June of the following year, consistent with previous studies (14), capturing the typical winter–spring peak of RSV activity.

### 2.4 Statistical analysis and modeling strategy

#### 2.4.1 Data preprocessing

To ensure data quality and model robustness, the following preprocessing procedures were implemented:

*Missing data imputation*: For meteorological variables with limited missing values (<5%). Missing data imputation was performed using a hybrid Seasonal-Trend decomposition based on Loess (STL) approach combined with Kalman smoothing to preserve both seasonal structure and temporal continuity of meteorological variables.

*Temporal aggregation*: RSV case data and meteorological data were aggregated by epidemiological week to align with the incubation period and reporting cycle of RSV. Weekly RSV positivity rates were calculated as the number of positive cases divided by the total number of tests. Monthly and annual aggregates were additionally generated for descriptive trend analysis.

*Collinearity assessment*: Spearman correlation coefficients were calculated among meteorological variables. To minimize multicollinearity, for variable pairs with |r| > 0.7, only the variable demonstrating a stronger association in univariate analysis was retained for subsequent modeling.

#### 2.4.2 Descriptive analysis and trend assessment

Temporal trends in monthly RSV positivity within each epidemic season were assessed using a Poisson regression model with a log link function and an offset term for the total number of tests. This framework effectively models changes in the proportion of positive tests over time. The model was specified as:


$$\log(\mu_{t})=\beta_{0}+\beta_{1}\mathrm{Tim}e_{t}+\log(\text{tota}l_{t})$$

where $\mu_{t}$ represents the expected number of RSV-positive cases, $\text{tota}l_{t}$ denotes the total number of tests, and $\mathrm{Tim}e_{t}$ indicates the sequential month within each epidemic season. In this formulation, $\beta_{1}$ reflects the log-linear change in RSV positivity rate over time within each season.

The monthly percent change (MPC) was calculated as:


$$(e^{\beta_{1}}-1)\times 100\%$$

with corresponding 95% confidence intervals derived from the standard error of $\beta_{1}$ . In addition, a non-parametric bootstrap procedure with 10,000 resamples was performed to obtain robust confidence intervals for the estimated percentage change, by repeatedly resampling observations within each epidemic season and refitting the model to generate an empirical distribution of the effect size.

#### 2.4.3 Distributed lag non-linear model

To quantify the non-linear exposure–response relationship and lag effects of meteorological factors on RSV transmission, a Distributed Lag Non-linear Model based on a Quasibinomial regression framework was constructed. The model specification was as follows:


$$\begin{array}{c} \text{Logit}(p_{t})=\beta_{0}+\mathrm{cb}(X_{t},\mathrm{lag})+\sum_{k}f(Z_{k,t})+\mathrm{ns}(\text{time},\mathrm{df})\\ +\text{factor}(\text{Stage})+\sin(\frac{2\mathrm{\pi t}}{52})+\cos(\frac{2\mathrm{\pi t}}{52}) \end{array}$$

where $\beta_{0}$ is the intercept; the RSV positivity probability in week t(t=1,2,3,…,209) was defined as $p_{t}=Y_{t}/n_{t}$ and modeled using a logit link function. The model incorporated a cross-basis function $\mathrm{cb}(X_{t},\mathrm{lag})$ to simultaneously capture the non-linear exposure–response relationship and lagged effects of the meteorological variable $X_{t}$ . Natural cubic spline functions were used to model the non-linear effects of other meteorological covariates $Z_{k,t}$ , denoted as $\sum_{k}f(Z_{k,t})$ . Long-term temporal trends were controlled using a natural spline function of time, ns(time,df) , while factor(Stage) accounted for differences across epidemic phases. Seasonal variation was further adjusted using harmonic terms with a 52-week periodicity, defined as sin(2πt/52) and cos(2πt/52) .

Model parameters, including maximum lag weeks and degrees of freedom, were selected based on goodness-of-fit criteria, guided by quasi-Akaike information criterion (QAIC) and quasi-Bayesian information criterion (QBIC), and supported by previous literature (17) and preliminary analyses. The maximum lag was ultimately set at 3 weeks. Detailed model specifications and parameter settings are provided in Supplementary Table S5. The median (P_50_) of each meteorological variable served as the reference level. Relative risks (RR) and 95% confidence intervals (CI) were estimated, and two-dimensional exposure-response surfaces and lag-response plots were generated.

#### 2.4.4 Predictive modeling and SHAP interpretation

Given the complex non-linear interactions and threshold effects between meteorological factors and RSV transmission, an Extreme Gradient Boosting (XGBoost) model was constructed to develop a predictive framework. SHAP (Shapley Additive Explanations) was applied to enhance interpretability.

##### 2.4.4.1 XGBoost model construction strategy

*Base_model*: Constructed using RSV positivity rates.

*Base_enhanced_model*: Built upon the Base Model with additional engineered features.

*Climate-integrated models*: Individual meteorological variables were separately incorporated into the Base_Enhanced_Model to construct a series of parallel sub-models, each evaluating the incremental contribution of a single meteorological factor to predictive performance.

*Model evaluation*: Model performance was assessed using the coefficient of determination (R^2^), mean squared error (MSE), root mean squared error (RMSE), mean absolute error (MAE), and mean absolute scaled error with lag-1 and seasonal (s52) reference (MASE_lag1, MASE_s52). Detailed definitions and calculation procedures for MASE_lag1 and MASE_s52 are provided in the Supplementary materials.

*Interpretability analysis*: SHAP values were computed to quantify each feature’s marginal contribution to predictions. Summary plots were used to identify key drivers and characterize their non-linear influence patterns.

##### 2.4.4.2 Data source and temporal stratification

Model development was based on 204 weekly observations after 5-lag feature construction. The dataset was strictly ordered in chronological sequence and partitioned without shuffling.

*Training set*: 142 weeks (approximately first 70% of the time series).

*Validation set*: 21 weeks (approximately next 10% of the series).

*Test set*: 41 weeks (final 20% of the series).

##### 2.4.4.3 XGBoost feature engineering and lagged variable construction

Feature engineering was constructed exclusively using historical observations to avoid data leakage.

###### 2.4.4.3.1 RSV-related features

*Lag features*: RSV_lag1, RSV_lag2, RSV_lag3, RSV_lag4, RSV_lag5.

*Moving averages*: RSV_ma3, RSV_ma5.

*Trend indicator*: RSV_trend = RSV_lag1 − RSV_lag5.

*Volatility indicator*: RSV_volatility = |RSV_lag1 − RSV_lag2| + |RSV_lag2 − RSV_lag3|.

###### 2.4.4.3.2 Meteorological variables

For each meteorological variable X(X = TEMP, DEWP, SLP, VISIB, WDSP, MXSPD, PRCP, TD), the following features were constructed:

*Lag features*: X_lag1 – X_lag5.

*Trend*: X_trend = X_lag1 − X_lag5.

*Volatility*: X_volatility = |X_lag1 − X_lag2| + |X_lag2 − X_lag3|.

###### 2.4.4.3.3 Risk-derived features (distribution-based)

Risk features were constructed using quartile-based thresholds derived exclusively from the training set:

*High risk*: max [0, X_lag1 − Q75 (X_lag1)].

*Low risk*: max [0, Q25 (X_lag1) − X_lag1].

These features were computed for all meteorological variables and consistently applied to validation and test sets using training-derived quantiles only.

###### 2.4.4.3.4 Stage variable

*Stage*: Stage_lag1—Stage_lag5.

##### 2.4.4.4 XGBoost model features

*Base_model*: RSV_lag1.

*Base_enhanced_model*: RSV_lag1–5, Stage_lag1–5, RSV_ma3, RSV_ma5, RSV_trend, RSV_volatility.

*Climate-integrated models*: Each climate-integrated model extends the Base_Enhanced model by adding one meteorological variable with: Lag1–5 features, Trend, Volatility, High-risk and Low-risk lag1 indicators. Each climate model evaluates the marginal contribution of a single meteorological factor.

##### 2.4.4.5 XGBoost Hyperparameter optimization

Hyperparameter tuning for the Base model was performed using a predefined grid search strategy. Model selection was based on minimizing RMSE. For all enhanced models, the final hyperparameters are reported in the Supplementary Table S6.

##### 2.4.4.6 XGBoost time-series cross-validation strategy

A time-series cross-validation strategy was applied for hyperparameter tuning of the Base model. Specifically, an initial training window comprising 70% of the training data was used, and a forecasting horizon of 15% was defined for each resampling iteration. The remaining data were used to generate multiple sequential rolling validation splits, ensuring that all observations after the initial window were progressively used as validation folds. No random shuffling was applied at any stage.

##### 2.4.4.7 ARIMA benchmark forecasting model

An autoregressive integrated moving average (ARIMA) model was developed as a classical statistical benchmark using the weekly RSV positivity rate series. Model development was based exclusively on historical RSV observations without incorporating meteorological variables or engineered predictors. Forecasting was performed using a rolling forecasting framework, in which the model was continuously updated with all available historical observations and used to generate one-step-ahead predictions throughout the test period. Model performance was evaluated using the same metrics as the XGBoost models. Detailed ARIMA model specifications are provided in the Supplementary Table S7.

#### 2.4.5 Statistical software

All statistical analyses and modeling procedures were conducted using R software (version 4.4.2). The R packages included *stats* (regression and statistical testing), *dlnm* (distributed lag nonlinear modeling), *xgboost* (gradient boosting modeling and SHAP analysis), *caret* (model training and evaluation framework), *forecast* (ARIMA modeling and forecasting), and *tseries* (stationarity testing). Stage-specific linear regression for temporal trend analysis was carried out using the *stats* package. SHAP values were computed using the TreeSHAP algorithm implemented in the *xgboost* package, and global feature importance was quantified as the mean absolute SHAP value across the test set. ARIMA modeling was implemented using the *forecast* package, and stationarity testing was performed using the *tseries* package. Rolling one-step-ahead forecasting was conducted through recursive model updating using all available historical observations. All statistical tests were two-sided, with *p-value* < 0.05 considered statistically significant.

## 3 Results

### 3.1 Characteristics of the study population

From July 1, 2019 to July 2, 2023, a total of 38,541 individuals underwent RSV testing, including 33,141 negative and 5,400 positive cases (Table 1).

**Table 1:** Characteristics of the study subjects.

Factor	Total cases (*n* = 38,541)		Negative cases (*n* = 33,141)		Positive cases (*n* = 5,400)		df	*χ^2^*	*p-value*
	*n*	%	*n*	%	*n*	%			
Sex									
Male	22,692	58.88	19,517	58.89	3,175	58.80	1	0.017	0.896
Female	15,849	41.12	13,624	41.11	2,225	41.20			
Age group									
1–5 months (young infants)	5,396	14.00	4,387	13.24	1,009	18.69	4	1024.759	< 0.0001^****^
6–24 months (older infants and toddlers)	15,820	41.05	12,909	38.95	2,911	53.91			
3–5 years (preschool children)	11,276	29.26	9,980	30.11	1,296	24.00			
6–17 years (school-age children and adolescents)	3,705	9.61	3,551	10.71	154	2.85			
18 + years (adult)	2,344	6.08	2,314	6.98	30	0.56			
Season									
Spring (Mar, Apr, May)	8,768	22.75	8,280	24.98	488	9.04	3	879.237	< 0.0001^****^
Summer (Jun, Jul, Aug)	8,120	21.07	7,059	21.30	1,061	19.65			
Autumn (Sep, Oct, Nov)	8,092	21.00	6,408	19.34	1,684	31.19			
Winter (Dec, Jan, Feb)	13,561	35.19	11,394	34.38	2,167	40.13			

*p-*value* < 0.05; **p-*value* < 0.01; ***p-*value* < 0.001; ****p-*value* < 0.0001.

Among positive cases, 3,175 (58.80%) were male and 2,225 (41.20%) were female. There was no statistically significant difference in sex distribution between positive and negative groups (*χ^2^* = 0.017, *p-value* = 0.896).

The overall case distribution was predominantly concentrated in young children. Older infants and toddlers (6–24 months) accounted for 53.91% of positive cases and represented the primary affected population, followed by young infants (1–5 months) (18.69%) and preschool children 3–5 years (24.00%). School-age children and adolescents (6–17 years) and adults (18+ years) accounted for 2.85 and 0.56%, respectively. Positivity rates differed significantly across age groups (*χ^2^* = 1024.759, *p-value* < 0.0001), with cases mainly occurring in children under 2 years of age.

Seasonal distribution also differed significantly (*χ^2^* = 879.237, *p-value* < 0.0001). Positive cases were more frequently observed in autumn (31.19%) and winter (40.13%). Although the proportional distribution fluctuated across epidemic seasons, autumn and winter consistently accounted for the highest case concentrations.

### 3.2 Seasonal patterns and epidemic period characteristics of RSV in Guangdong, 2019–2023

**Figure 1:**
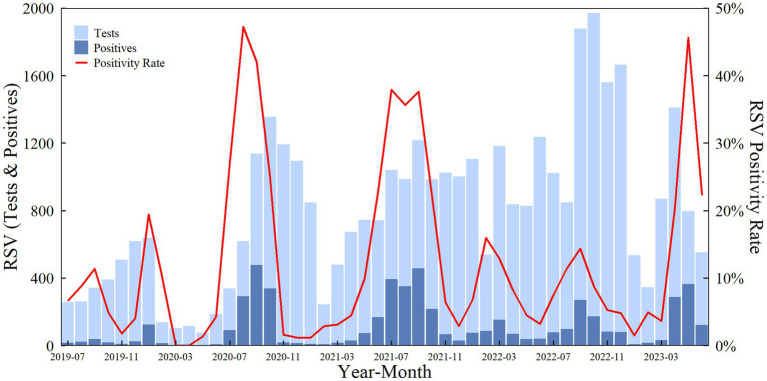
Temporal trends in monthly RSV positivity rate in Guangdong Province, 2019–2023.

Between July 2019 and July 2023, monthly RSV positivity rates in Guangdong exhibited cyclical fluctuations (Figure 1). Weekly RSV positivity rates exhibited clear cyclical fluctuations throughout the study period (Supplementary Figure S2). Using the four-year mean positivity rate of 12.62% as the reference, several periods exceeded this baseline, including January 2020; July–October 2020; June–October 2021; February–March 2022; September 2022; and April–June 2023. Notably, after July 2020, elevated positivity rates persisted across consecutive months, forming cross-year epidemic peaks. A renewed increase was observed during April–June 2023. The timing of peak months varied across epidemic seasons, and several peaks were no longer confined to the traditional winter–spring period.

Based on the definition of the shortest consecutive interval accounting for 80% of cumulative seasonal RSV positivity rate, the epidemic periods were as follows (Table 2): 2019/2020 season (August 2019–February 2020): 7 months; 2020/2021 season (July 2020–March 2021): 9 months; 2021/2022 season (July 2021–February 2022): 8 months; 2022/2023 season (August 2022–May 2023): 10 months.

**Table 2:** MPC cumulative positivity rate: thresholds and temporal characteristics.

Epidemic season	Threshold	Start and end (year-month)	Duration (months)
2019/2020	75%	2019–07 ~ 2020–01	7
	80%	2019–08 ~ 2020–02	7
	85%	2019–07 ~ 2020–02	8
2020/2021	75%	2020–07 ~ 2020–11	5
	80%	2020–07 ~ 2021–03	9
	85%	2020–07 ~ 2021–05	11
2021/2022	75%	2021–07 ~ 2022–01	7
	80%	2021–07 ~ 2022–02	8
	85%	2021–07 ~ 2022–02	8
2022/2023	75%	2022–10 ~ 2023–06	9
	80%	2022–08 ~ 2023–05	10
	85%	2022–09 ~ 2023–06	10

Sensitivity analyses using 75 and 85% thresholds yielded similar epidemic patterns. Epidemic durations generally exceeded 6 months, except for the 2020/2021 season under the 75% threshold (5 months), which likely reflected the substantial disruption of RSV circulation during the COVID-19 pandemic. The 2022/2023 season showed the longest epidemic duration. Overall, RSV epidemic periods demonstrated prolongation and cross-seasonal distribution during the study period.

**Figure 2:**
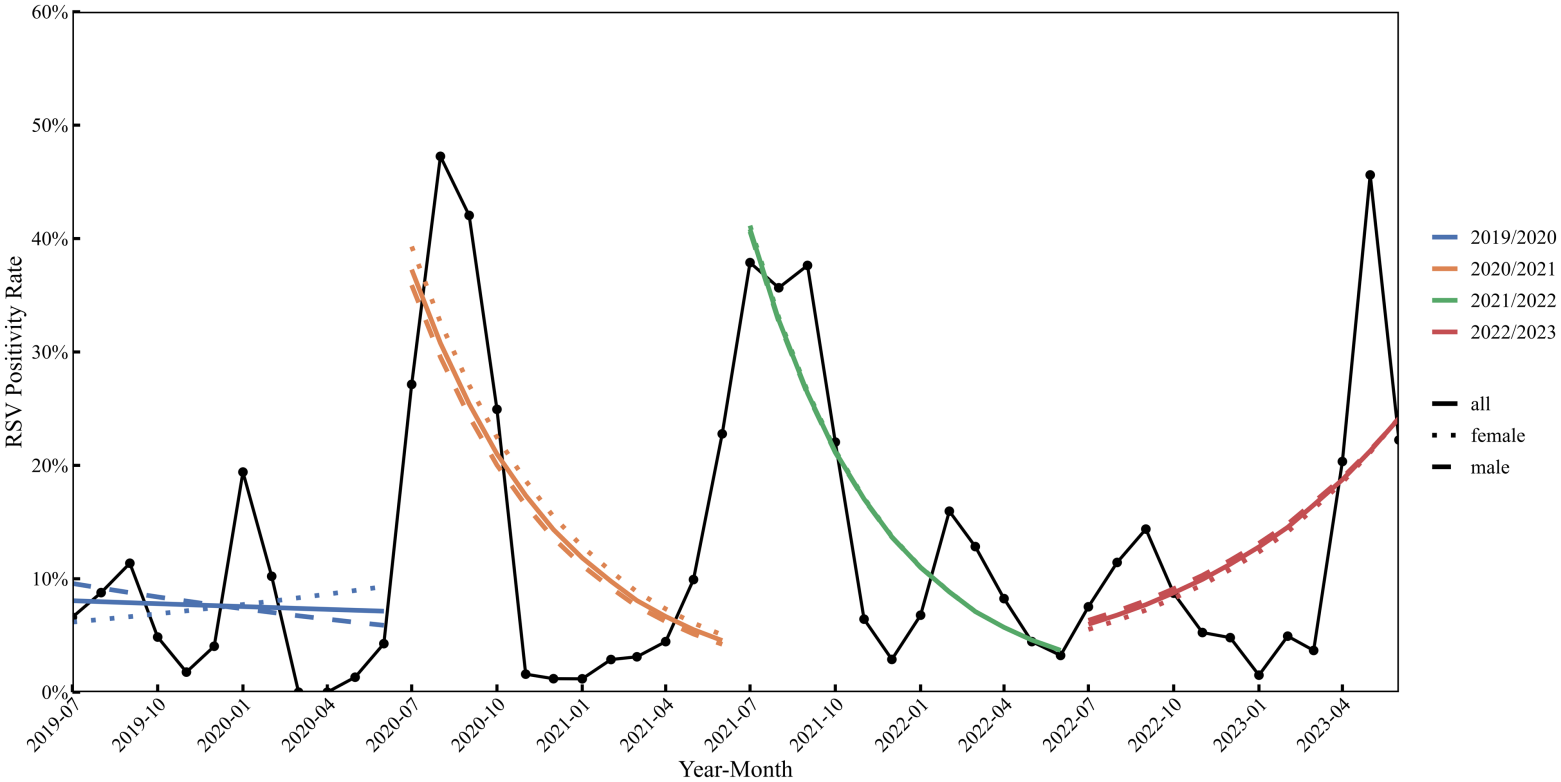
Temporal trends of RSV positivity rates across epidemic seasons in Guangdong Province, 2019–2023.

Stage-specific linear regression analysis of RSV positivity rates from July 2019 to June 2023 revealed stage-specific temporal trends (Figure 2).

During 2019/2020 (July 2019–June 2020), the overall RSV positivity rate showed a decreasing trend, with a monthly percentage change (MPC) of −1.10% (95% CI: −5.24, 3.22%). In 2020/2021, the MPC was −17.39% (95% CI: −18.95, −15.80%), indicating a continued decline. A similar strong decreasing trend persisted in 2021/2022, the downward trend persisted, with an MPC of −19.62% (95% CI: −20.83, −18.39%). In contrast, during 2022/2023 (July 2022–June 2023), the MPC increased to 13.51% (95% CI: 11.84, 15.21%), suggesting an upward trend. Notably, the marked declines observed in 2020/2021 and 2021/2022, followed by a rebound in 2022/2023, suggested a potential shift in epidemic timing, with earlier seasonal decline phases in 2020/2021–2021/2022 and a delayed resurgence pattern in 2022/2023. Similar temporal patterns were observed in sex-stratified analyses, with both females and males exhibiting trends consistent with the overall population across epidemic seasons (Table 3).

**Table 3:** Seasonal and sex-specific MPC of RSV positivity rate.

Epidemic Season	Group	MPC (%)	95% CI (%)	*p*-*value*
2019/2020	All	−1.10	(−5.24, 3.22)	0.6140
	Female	3.79	(−2.85, 10.88)	0.2700
	Male	−4.32	(−9.55, 1.21)	0.1230
2020/2021	All	−17.39	(−18.95, −15.8)	0.0000^****^
	Female	−17.01	(−19.36, −14.59)	0.0000^****^
	Male	−17.68	(−19.75, −15.55)	0.0000^****^
2021/2022	All	−19.62	(−20.83, −18.39)	0.0000^****^
	Female	−19.69	(−21.58, −17.75)	0.0000^****^
	Male	−19.58	(−21.15, −17.98)	0.0000^****^
2022/2023	All	13.51	(11.84, 15.21)	0.0000^****^
	Female	14.36	(11.71, 17.07)	0.0000^****^
	Male	12.92	(10.76, 15.12)	0.0000^****^

95% confidence intervals (95% CI) were derived using the normal approximation of the regression coefficient.

*p-value < 0.05; **p-value < 0.01; ***p-value < 0.001; ****p-value < 0.0001.

Taken together, RSV activity in Guangdong between 2019 and 2023 was characterized by multiple episodic high-activity months, epidemic durations ranging from 7 to 10 months, and variability in onset timing and duration across seasons.

### 3.3 Comparison of RSV positivity rates across COVID-19 stages

The overall RSV positivity rate increased progressively across the three COVID-19 stages, rising from 8.19% in Stage I to 13.84% in Stage II and 19.33% in Stage III. Pairwise comparisons demonstrated significant differences between Stage I and Stage II (*χ^2^* = 74.726, *p-value* < 0.0001), Stage I and Stage III (*χ^2^* = 172.655, *p-value* < 0.0001), and Stage II and Stage III (*χ^2^* = 91.147, *p-value* < 0.0001).

Stratified by sex, male positivity rates increased from 8.76 to 13.74% and further to 19.46%. The difference between Stage I and Stage II was not statistically significant (*χ^2^* = 33.791, *p-value* < 0.0001), whereas comparisons between Stage I and Stage III (*χ^2^* = 91.361, *p-value* < 0.0001) and between Stage II and Stage III (*χ^2^* = 58.402, *p-value* < 0.0001) were significant. Among females, positivity rates increased from 7.37 to 13.98% and then to 19.15%, with significant differences across all pairwise stage comparisons (*p-value* < 0.0001).

Age-stratified analyses showed significant differences in RSV positivity rates across three stages in young infants (*P. value* = 0.0007), older infants and toddlers (*P. value* < 0.0001), and preschool children (*p-value* < 0.0001), whereas no significant differences were observed in the school-age children and adolescents (*P. value* = 0.2347). In adults, a marginal difference was detected (*p-value* = 0.0388).

Pairwise comparisons across epidemic stages further demonstrated that in young infants, a significant difference was observed only between Stage II and Stage III, while no significant differences were detected between Stage I and Stage II or between Stage I and Stage III. In contrast, older infants and toddlers, and preschool children showed significant differences across all stage comparisons, with consistently higher positivity rates in Stage III. In adults, no statistically significant differences were observed after pairwise comparisons.

Overall, stage-related changes in RSV positivity were primarily driven by children under 6 years of age, whereas older age groups remained relatively stable across epidemic stages (Table 4).

**Table 4:** Comparison and distribution of RSV positivity rate across different COVID-19 stage.

Parameter	Stage I (Pre-COVID-19 pandemic)	Stage II (COVID-19 pandemic)	Stage III (Post-pandemic)	*χ^2^* (I vs II)	*p-value*	*χ^2^* (I vs III)	*p-value*	*χ* (II vs III)	*p-value*
	*n* (%)	*n* (%)	*n* (%)						
Total	244 (8.19)	4,329 (13.84)	827 (19.33)	74.726	0.0000^****^	172.655	0.0000^****^	91.147	0.0000^****^
Gender									
Male	153 (8.76)	2,531 (13.74)	491 (19.46)	33.791	0.0000^****^	91.361	0.0000^****^	58.402	0.0000^****^
Female	91 (7.37)	1798 (13.98)	336 (19.15)	41.901	0.0000^****^	81.170	0.0000^****^	32.596	0.0000^****^
Age									
1–5 months	38 (20.43)	856 (18.01)	115 (25.22)	0.556	1.0000	1.416	0.7022	13.806	0.0006^***^
6–24 months	146 (10.38)	2,344 (18.10)	421 (28.84)	52.228	0.0000^****^	152.717	0.0000^****^	96.918	0.0000^****^
3–5 years	50 (5.91)	996 (10.91)	250 (19.25)	20.077	0.0000^****^	74.631	0.0000^****^	74.363	0.0000^****^
6–17 years	9 (2.47)	114 (4.31)	31 (4.45)	*χ^2^* = 2.899, *p-value* = 0.2347					
18 + years	1 (0.56)	19 (1.06)	10 (2.73)	—	1.0000	—	0.3376	—	0.0621

*χ^2^* and *p-values* were obtained from Pearson’s chi-square test or Fisher’s exact test, as appropriate. Bonferroni correction was applied for pairwise comparisons.

*p-*value* < 0.05; **p-*value* < 0.01; ***p-*value* < 0.001; ****p-*value* < 0.0001.

### 3.4 Meteorological characteristics and correlation with RSV positivity, 2019–2023

**Figure 3:**
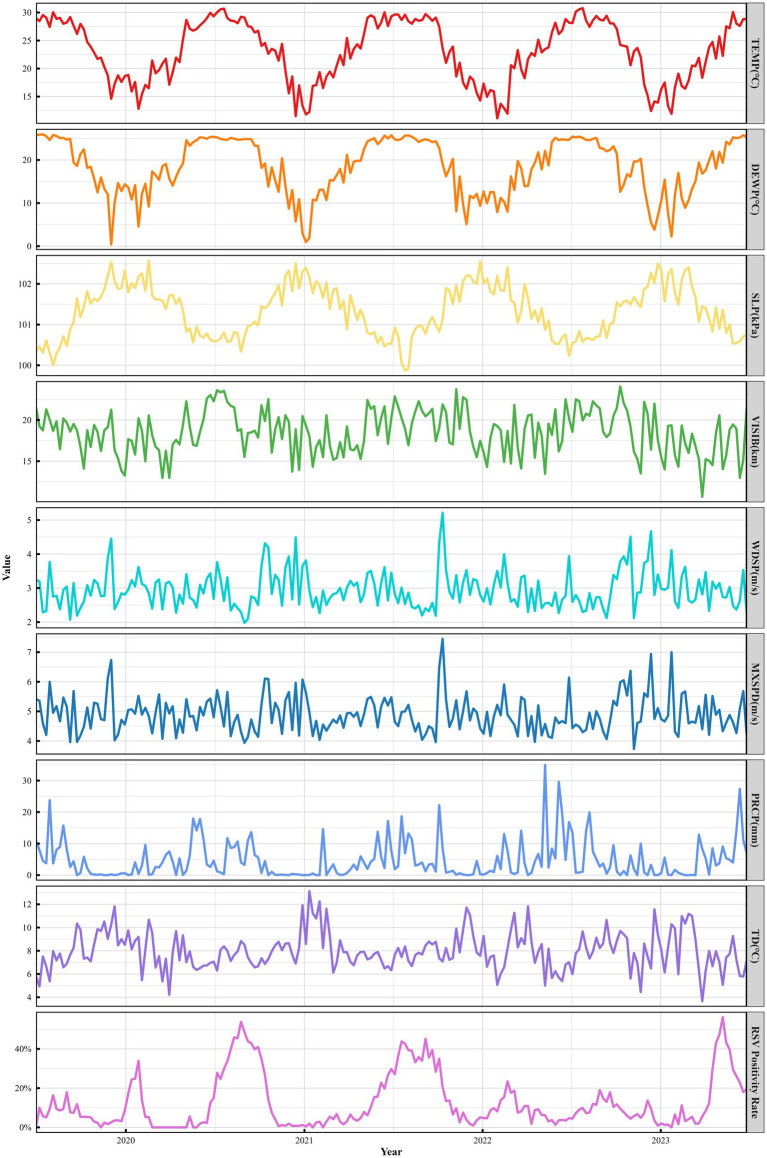
Time-series patterns of meteorological factors in Guangdong Province, 2019–2023.

From July 2019 to July 2023, meteorological variables in Guangdong displayed stable seasonal patterns (Figure 3; Supplementary Table S4). The mean weekly RSV positivity rate during the study period was 12.72%. Median temperature and dew point temperature were 23.67 °C and 19.20 °C, respectively, showing higher values in summer and lower values in winter. Mean sea-level pressure (P_50_ = 101.32 kPa) was higher in winter and lower in summer. Median precipitation was 2.60 mm, with greater variability in summer. Median wind speed and maximum sustained wind speed were 2.84 m/s and 4.82 m/s, respectively, showing relatively stable variation.

Spearman correlation analysis (Supplementary Figure S1) demonstrated positive correlations between RSV positivity rate and temperature (*r* = 0.47, *p-value* < 0.0001), dew point temperature (*r* = 0.44, *p-value* < 0.0001), visibility (*r* = 0.28, *p-value* < 0.0001), and precipitation (*r* = 0.34, *P. value* < 0.0001). Mean sea-level pressure was negatively correlated with RSV positivity rate (*r* = −0.42, *p-value* < 0.0001).

Strong correlations were observed among several meteorological variables, including temperature and dew point temperature (r = 0.94), temperature and mean sea-level pressure (*r* = −0.90), and dew point temperature and mean sea-level pressure (*r* = −0.94) (all *p-value* < 0.0001). Wind speed and maximum sustained wind speed were also highly correlated (*r* = 0.92, *p-value* < 0.0001).

### 3.5 Non-linear exposure-response relationships and lag effects of meteorological drivers

**Figure 4:**
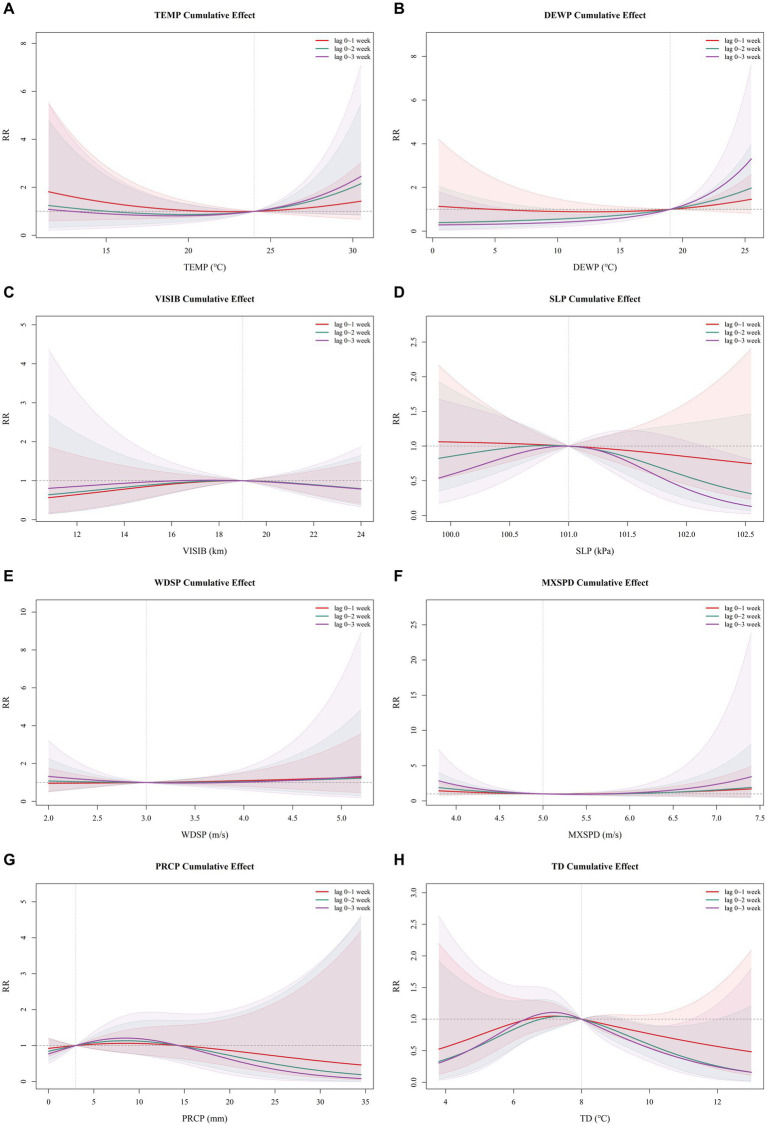
**(A)** Cumulative effect of temperature over lag weeks 0–3. **(B)** Cumulative effect of dew point over lag weeks 0–3. **(C)** Cumulative effect of visibility over lag weeks 0–3. **(D)** Cumulative effect of sea-level pressure over lag weeks 0–3. **(E)** Cumulative effect of wind speed over lag weeks 0–3. **(F)** Cumulative effect of maximum wind speed over lag weeks 0–3. **(G)** Cumulative effect of precipitation over lag weeks 0–3. **(H)** Cumulative effect of temperature difference over lag weeks 0–3.

Using the median of each meteorological variable as the reference, DLNM analysis demonstrated heterogeneous and non-linear exposure–response relationships between RSV positivity rates and meteorological factors (Figure 4). Detailed two-dimensional contour visualizations of the exposure–lag–response associations are provided in Supplementary Figure S3.

The associations were stratified into four categories based on effect magnitude, non-linear shape consistency, and statistical stability: Strong variables (TEMP, DEWP, WDSP, MXSPD, PRCP) exhibited clear non-linear exposure–response patterns; TD showed moderately consistent associations; VISIB demonstrated weak but relatively structured associations with a narrow exposure window associated with increased RSV positivity rate risk; SLP showed unstable associations across lag structures.

Across lag 1–3 weeks, most meteorological variables (TEMP, DEWP, WDSP, MXSPD, PRCP, TD, and VISIB) exhibited localized exposure ranges associated with increased RSV positivity risk (RR > 1.00), with substantial overlap of risk windows across lag structures. Specifically, elevated risk was observed for TEMP (11.5–12.5 °C and 24.0–30.5 °C), DEWP (19.0–25.5 °C), WDSP (3.90–5.20 m/s), MXSPD (3.8–5.0 m/s and 5.9–7.4 m/s), PRCP (3.0–14.0 mm), TD (6.6–8.0 °C), and VISIB (18.6–19.0 km), although VISIB showed a relatively narrow and less stable exposure window.

In contrast, SLP demonstrated a temporally unstable pattern, with positive associations observed at lag 1–2 weeks (100.70–101.00 kPa) but not sustained at lag 3 weeks, indicating non-persistent exposure–response behavior.

### 3.6 Performance evaluation and feature attribution of the XGBoost-SHAP predictive framework

**Figure 5:**
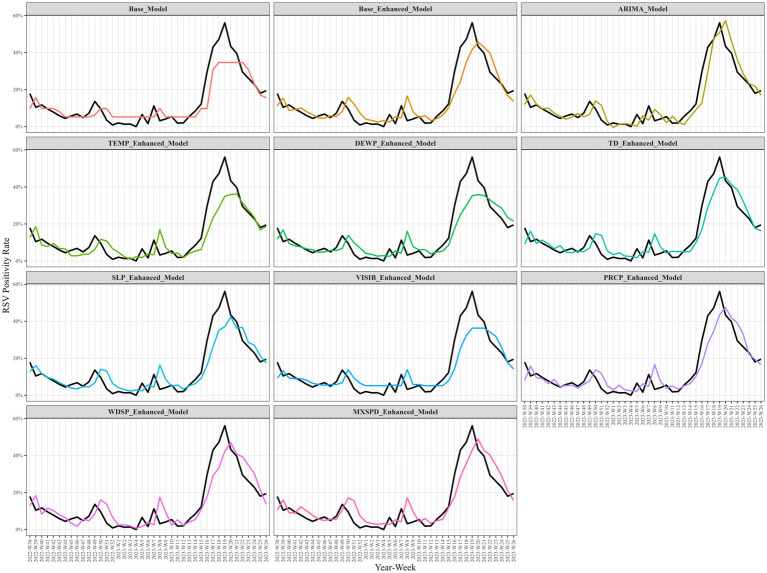
Test set predictions of models.

Predicted results of the XGBoost models on the test dataset are presented in Figure 5. All models captured temporal fluctuations and major inflection points in RSV positivity rates. Model performance metrics are summarized in Table 5.

**Table 5:** Test set performance evaluation of models.

Model	*R^2^*	MAE	MSE	RMSE	MASE_lag1	MASE_s52
Base_model	0.8333	0.0452	0.0043	0.0654	1.2566	0.4302
Base_enhanced_model	0.8078	0.0467	0.0040	0.0631	1.2988	0.4446
TEMP_enhanced_model	0.7898	0.0491	0.0052	0.0722	1.3666	0.4678
DEWP_enhanced_model	0.7930	0.0497	0.0048	0.0689	1.3820	0.4731
SLP_enhanced_model	0.8132	0.0451	0.0041	0.0638	1.2551	0.4296
VISIB_enhanced_model	0.8176	0.0483	0.0045	0.0670	1.3440	0.4601
PRCP_enhanced_model	0.8248	0.0443	0.0036	0.0600	1.2324	0.4219
WDSP_enhanced_model	0.7919	0.0505	0.0042	0.0651	1.4051	0.4810
MXSPD_enhanced_model	0.7971	0.0507	0.0042	0.0647	1.4101	0.4827
TD_enhanced_model	**0.8405**	**0.0441**	**0.0033**	**0.0573**	**1.2261**	**0.4197**
ARIMA_model	0.8581	0.0410	0.0031	0.0559	1.1412	0.3907

Underlined values denote the best-performing model in each category.

Bolded values indicate the best-performing models with the inclusion of meteorological predictors.

The Base_Model achieved an R^2^ of 0.8333, RMSE of 0.0654, and MAE of 0.0452 on the test set. After feature engineering, the Base_Enhanced_Model showed a slight decrease in explanatory power (*R^2^* = 0.8078), while achieving a reduction in RMSE (0.0631), although MAE slightly increased to 0.0467.

Among all climate-integrated models, the TD_Enhanced_Model demonstrated the best performance, with a test-set *R^2^* of 0.8405, RMSE of 0.0573, and MAE of 0.0441, outperforming both the Base_Model and Base_Enhanced_Model. The SLP_Enhanced_Model, VISIB_Enhanced_Model, and PRCP_Enhanced_Model showed intermediate performance, with R^2^ values of 0.8132, 0.8176, and 0.8248, respectively. These models outperformed the Base_Enhanced_Model but remained inferior to the Base_Model. In contrast, all remaining climate-integrated models demonstrated lower *R^2^* values than both baseline models. Furthermore, ARIMA_Model achieved an *R^2^* of 0.8581, RMSE of 0.0559, and MAE of 0.0410, ranking among the best-performing models in terms of overall predictive accuracy.

Single-variable gain analysis showed that inclusion of TD yielded the largest improvement, increasing *R^2^* by approximately 0.072 compared with the Base_Model. Although the inclusion of SLP, VISIB, and PRCP did not improve predictive performance over the Base_Model, they yielded modest but consistent gains compared with the Base_Enhanced_Model, suggesting limited incremental predictive value beyond feature engineering alone.

Across all models, performance relative to naïve benchmarks was evaluated using MASE_lag1 and MASE_s52. MASE_lag1 values greater than 1 indicated that most models did not consistently outperform short-term persistence-based forecasting, whereas MASE_s52 values below 1 suggested improved performance relative to seasonal naïve forecasting, reflecting stronger capture of seasonal structure than short-term autocorrelation patterns.

Across all models, MASE_lag1 values exceeded 1, indicating limited improvement over short-term persistence-based forecasting and suggesting that short-term fluctuations in RSV positivity were difficult to capture. In contrast, MASE_s52 values were consistently below 1, demonstrating better performance relative to seasonal naïve forecasting and indicating that seasonal patterns were more stable and predictable than short-term variations.

Overall, these findings suggest that RSV positivity rates exhibit pronounced seasonal structure alongside complex short-term variability that is less predictable and may be influenced by external factors not explicitly captured by the models.

**Figure 6:**
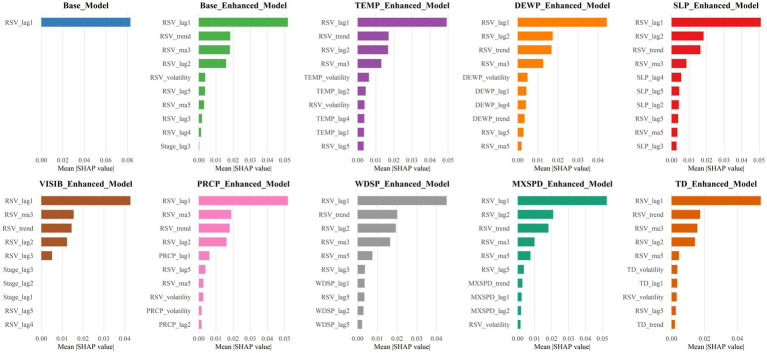
SHAP feature importance across XGBoost models.

SHAP feature importance results are shown in Figure 6. Lagged RSV-derived features consistently dominated model interpretation across all XGBoost models. RSV_lag1 appeared in all models, and ranked as the most frequently selected feature, followed by RSV_lag2, RSV_ma3, and RSV_trend, indicating that intrinsic temporal dependence of RSV positivity rate constituted the primary predictive structure.

Consistent with feature selection frequency, SHAP-based importance analysis further confirmed that RSV_lag1 was the most influential predictor across all models, with the highest mean absolute SHAP values in each model variant. Other RSV-derived features, including RSV_lag2, RSV_trend, and RSV_ma3, also consistently ranked among the top contributors, reinforcing the dominant role of autoregressive and short-term memory effects in RSV dynamics.

Meteorological variables provided additional but relatively limited predictive information. Across climate-augmented models, PRCP, SLP, and DEWP exhibited moderate SHAP importance and were occasionally ranked among top contributors, although their effects were consistently weaker than those of RSV-derived predictors.

However, the inclusion of VISIB led to only minor changes in model performance compared with the Base_Enhanced_Model, suggesting limited incremental predictive value. This effect likely reflects the weak marginal signal of visibility in RSV dynamics, as well as structural perturbations introduced by tree-based model re-fitting when additional features are incorporated.

## 4 Discussion

This study systematically characterized the epidemiological patterns of RSV in Guangdong Province from 2019 to 2023 and examined its climate-driven determinants. We observed a post-pandemic reconfiguration of RSV seasonality, with prolonged epidemic periods and cross-seasonal distribution. Meteorological variables were associated with RSV activity and demonstrated non-linear threshold effects and short-term lag effects in the DLNM analysis. Furthermore, the interpretable predictive framework based on XGBoost-SHAP showed stable performance in trend forecasting. Collectively, these findings delineate new features of RSV transmission in the post-COVID-19 era from the perspectives of epidemiological restructuring, environmental drivers, and predictive modeling.

Consistent with previous evidence, RSV exhibits distinct regional seasonality across different climatic zones. A global systematic analysis by Li et al. reported that RSV activity in tropical and subtropical regions tends to be more dispersed, often presenting multiple peaks or extended epidemic periods (18). Studies conducted in Chongqing also identified associations between meteorological factors and RSV transmission (17). During the COVID-19 pandemic, several countries reported earlier onset or off-season RSV outbreaks (19), and similar post-policy resurgence patterns were observed across multiple regions in China (20–22). The prolonged and cross-seasonal epidemic patterns identified in Guangdong align with these global and domestic observations but appear more pronounced in duration, suggesting that subtropical climatic conditions and high population mobility may have contributed to the restructuring of epidemic dynamics.

At the level of climate-driven mechanisms, this study identified non-linear exposure–response relationships between RSV activity and temperature, dew point temperature, and atmospheric pressure. Previous studies have demonstrated that temperature and humidity substantially influence RSV activity (18, 23). Potential mechanisms include alterations in viral survival and aerosol dynamics under varying thermal and humidity conditions, as well as impacts on host respiratory mucosal barriers (24). Atmospheric pressure changes may influence viral transmission through modifications in air density and particle suspension stability (25). The DLNM analysis further indicated that climate-related effects were concentrated within short-term lag windows, consistent with prior reports describing delayed environmental influences on respiratory virus transmission (17, 23). These findings suggest that meteorological exposures affect infection risk over a defined temporal window rather than exerting immediate effects. However, During the COVID-19 pandemic, healthcare-seeking behavior, clinical testing practices, healthcare accessibility, and public awareness of respiratory infections likely changed over time. Consequently, differences in RSV positivity observed across pandemic stages may partially reflect changes in testing patterns in addition to underlying changes in RSV circulation. While positivity rate is a commonly used indicator in laboratory-based surveillance studies, it should be interpreted as a measure of RSV detection activity within the tested population rather than a direct measure of community transmission or disease incidence.

An important observation was the partial inconsistency between the DLNM and XGBoost findings. Temperature exhibited relatively strong nonlinear exposure–response relationships and lag effects in the DLNM analysis, yet its inclusion did not improve predictive performance in the XGBoost models. In contrast, precipitation showed localized risk associations in the DLNM and contributed modest predictive gains in some machine-learning models. These differences likely reflect the distinct objectives of the two analytical frameworks. DLNM evaluates the magnitude and temporal structure of associations between meteorological exposures and RSV activity, whereas XGBoost assesses the incremental contribution of variables to prediction after accounting for strong autoregressive signals. Consequently, variables that exhibit statistically meaningful associations may provide limited additional predictive value when their information is already captured by lagged RSV indicators, seasonal patterns, or correlated meteorological variables. Conversely, variables with relatively modest marginal associations may still improve prediction by contributing nonlinear interactions or complementary information not represented in autoregressive features. These findings underscore the distinction between explanatory and predictive modeling and suggest that the epidemiological relevance of a meteorological factor should not be inferred solely from its predictive importance.

From a methodological perspective, this study integrated DLNM with an XGBoost-SHAP framework. DLNM enabled quantification of non-linear threshold effects and lag structures, enhancing the characterization of climate–disease associations. The machine learning model improved predictive accuracy and employed SHAP (Shapley Additive Explanations) to quantify variable contributions (26). This integrated approach balances statistical inference with interpretability, providing methodological support for the development of climate-driven early warning frameworks for infectious diseases. Previous studies have highlighted the potential of machine learning for respiratory virus prediction (23), and the present analysis validates its feasibility within a regional surveillance context.

From a public health perspective, the observed prolongation of epidemic periods and seasonal restructuring suggest that intervention strategies based solely on traditional winter–spring RSV peaks may not adequately reflect current transmission dynamics. Because the study period ended in June 2023, before RSV vaccines and long-acting monoclonal antibodies were available for routine use in mainland China, the implications of our findings for these preventive measures should be considered prospective. As RSV immunization strategies continue to evolve and become increasingly available, a better understanding of local epidemic timing, seasonal variability, and climate-related drivers may help inform future implementation planning and timing of preventive interventions. In addition, climate-informed surveillance and forecasting approaches may support healthcare resource allocation and preparedness during periods of elevated RSV activity. The resurgence of respiratory viruses in the post-pandemic period further highlights the importance of sustained surveillance and continuous evaluation of changing transmission patterns (20, 27).

Several limitations should be acknowledged. First, this study was based on a laboratory-referred testing population rather than a population-based surveillance system. Although specimens were submitted from hospitals across multiple regions of Guangdong Province and included institutions of different healthcare levels, the tested population may not fully represent the general population. Second, social behavior and population mobility variables were not incorporated, precluding comprehensive assessment of non-climatic drivers. Third, the generalizability of the predictive model requires validation in other regions and over longer time horizons. Future studies should integrate multicenter surveillance data and multidimensional exposure variables to enhance model robustness and external validity.

## 5 Conclusion

RSV epidemiology in Guangdong from 2019 to 2023 exhibited seasonal reconfiguration and prolonged epidemic activity during the post-pandemic period. Meteorological factors showed nonlinear threshold relationships and short-term lag effects with RSV activity, indicating important climate-related influences on transmission dynamics. The combined DLNM and XGBoost-SHAP framework identified interpretable climate-risk patterns and captured major temporal trends in RSV activity. While predictive accuracy varied across epidemiological phases, particularly during periods of low activity and rapid transitions, the framework may support climate-informed surveillance and trend forecasting.

## Data Availability

The data analyzed in this study is subject to the following licenses/restrictions: the datasets supporting the conclusions of this article are included within the article and its Supplementary material. Additional resources are available from the corresponding author upon reasonable request. Requests to access these datasets should be directed to JC chenjb@gzhmu.edu.cn.
