# Supplementary material for: Epidemiological patterns and trends of respiratory syncytial virus in Guangdong Province, China, 2019–2023
Source: Front Public Health. 2026 Jul 31;14:1851186. doi: 10.3389/fpubh.2026.1851186 (PMC13473369; doi:10.3389/fpubh.2026.1851186)
Supplement: Supplementary file 1 [file Supplementary_File_1.docx]

Supplementary Material

# 1 Supplementary Data

## 1.1 Geographic distribution of RSV specimens in the study

**Supplementary Table S1. Geographic distribution of RSV specimens in the study**

| Region | Proportion(%) |
| --- | --- |
| Dongguan City | 0.236702868 |
| Foshan City | 35.39404066 |
| Guangzhou City | 13.35282651 |
| Jiangmen City | 0.006961849 |
| Jieyang City | 1.416736285 |
| Maoming City | 0.024366472 |
| Qingyuan City | 0.055694793 |
| Shantou City | 2.847396268 |
| Shaoguan City | 0.087023113 |
| Shenzhen City | 42.1679198 |
| Yangjiang City | 0.045252019 |
| Yunfu City | 0.003480925 |
| Zhanjiang City | 0.013923698 |
| Zhaoqing City | 0.452520189 |
| Zhongshan City | 0.11487051 |
| Zhuhai City | 3.780284043 |

## 1.2 Meteorological data

### 1.1.1 Data Transformation

To ensure consistency in measurement units, meteorological variables were converted as follows:

(1) Temperature variables (TEMP, DEWP, MAX, and MIN): ℃ = (°F - 32) / 1.8

(2) Pressure variables (SLP): kPa = bar / 10

(3) Visibility (VISIB): km = mi * 1.609344

(4) Wind speed variables (WDSP and MXSPD): m/s = knots * 0.5144444

(5) Precipitation (PRCP): mm = inch * 25.4

### 1.1.2 Missing data

There were 3 days of missing data (2021-02-11, 2021-02-12, and 2021-02-20).

### 1.1.3 Missing Data Imputation

Missing values were imputed using the Seasonal-Trend decomposition based on Loess (STL) combined with Kalman smoothing, implemented in R. The imputation procedure was performed using the entire time series from July 1, 2019 to July 2, 2023 to preserve the underlying seasonal and temporal structure of the data.

### 1.1.4 Comparison of Data Characteristics Before and After Imputation

**Supplementary Table S2. Data Characteristics Before Imputation**

| Variable | Min | P_25_ | Median | Mean | P_75_ | Max |
| --- | --- | --- | --- | --- | --- | --- |
| TEMP(℃) | 6.36 | 18.83 | 24.00 | 23.19 | 28.17 | 31.85 |
| DEWP(℃) | -6.72 | 13.71 | 19.64 | 18.38 | 24.66 | 26.62 |
| SLP(kPa) | 99.64 | 100.77 | 101.32 | 101.32 | 101.83 | 102.96 |
| VISIB(km) | 6.40 | 16.21 | 18.80 | 18.53 | 21.33 | 25.28 |
| WDSP(m/s) | 1.63 | 2.33 | 2.78 | 2.95 | 3.37 | 7.52 |
| MXSPD(m/s) | 2.62 | 4.14 | 4.71 | 4.90 | 5.42 | 10.00 |
| MAX(℃) | 8.59 | 23.83 | 28.46 | 27.65 | 32.47 | 36.87 |
| MIN(℃) | 2.65 | 14.90 | 20.62 | 19.66 | 25.09 | 28.07 |
| PRCP(mm) | 0.00 | 0.01 | 0.82 | 4.66 | 5.65 | 64.92 |

**Supplementary Table S3. Data Characteristics After Imputation**

| Variable | Min | P_25_ | Median | Mean | P_75_ | Max |
| --- | --- | --- | --- | --- | --- | --- |
| TEMP(℃) | 6.36 | 18.83 | 24.00 | 23.19 | 28.17 | 31.85 |
| DEWP(℃) | -6.72 | 13.71 | 19.64 | 18.38 | 24.66 | 26.62 |
| SLP(kPa) | 99.64 | 100.77 | 101.32 | 101.32 | 101.83 | 102.96 |
| VISIB(km) | 6.40 | 16.21 | 18.80 | 18.53 | 21.33 | 25.28 |
| WDSP(m/s) | 1.63 | 2.33 | 2.78 | 2.95 | 3.37 | 7.52 |
| MXSPD(m/s) | 2.62 | 4.14 | 4.71 | 4.90 | 5.42 | 10.00 |
| MAX(℃) | 8.59 | 23.83 | 28.46 | 27.65 | 32.47 | 36.87 |
| MIN(℃) | 2.65 | 14.90 | 20.62 | 19.66 | 25.09 | 28.07 |
| PRCP(mm) | 0.00 | 0.01 | 0.82 | 4.66 | 5.65 | 64.92 |

The results before and after imputation were highly consistent, indicating that the imputation procedure had a negligible impact on the overall data distribution.

### 1.1.5 Data Processing

The diurnal temperature range (TD, °C) was calculated as the difference between the maximum and minimum temperatures:

$$\boldsymbol{TD}\mathbf{=}\boldsymbol{MAX}\mathbf{-}\boldsymbol{MIN}$$

After the derivation of TD, the original MAX and MIN variables were excluded from subsequent analyses.

All variables were then aggregated to the weekly level by calculating their mean values within each week.

**Supplementary Table S4. Summary Statistics of the Final Analytical Dataset (Weekly)**

| Variable | Min | P25 | Median | Mean | P75 | Max |
| --- | --- | --- | --- | --- | --- | --- |
| TEMP(℃) | 11.12 | 18.67 | 23.67 | 23.17 | 28.19 | 30.77 |
| DEWP(℃) | 0.40 | 13.65 | 19.20 | 18.37 | 24.69 | 25.95 |
| SLP(kPa) | 99.87 | 100.77 | 101.40 | 101.32 | 101.83 | 102.57 |
| VISIB(km) | 10.68 | 16.63 | 18.71 | 18.53 | 20.57 | 24.07 |
| WDSP(m/s) | 1.98 | 2.56 | 2.84 | 2.95 | 3.25 | 5.22 |
| MXSPD(m/s) | 3.73 | 4.43 | 4.82 | 4.90 | 5.25 | 7.45 |
| TD(℃) | 3.66 | 6.96 | 7.87 | 7.99 | 8.81 | 13.13 |
| PRCP(mm) | 0.00 | 0.31 | 2.60 | 4.68 | 6.41 | 34.94 |
| RSV | 0.00% | 3.01% | 7.45% | 12.72% | 17.88% | 56.14% |

## 1.2 Distributed Lag Non-linear Model Specification

The cross-basis functions and covariate specifications used in the models for each meteorological factor are summarized below. Model parameters were selected through a grid-search procedure by jointly considering the quasi-Akaike information criterion (QAIC) and quasi-Bayesian information criterion (QBIC). Preference was given to more parsimonious models when improvements in QAIC were marginal (< 2 units) while QBIC indicated a better fit (> 2-unit reduction).

To address multicollinearity, in highly correlated variable pairs (TEMP–DEWP, TEMP–SLP, DEWP–SLP and WDSP–MXSPD), we retained TEMP over DEWP and WDSP over MXSPD based on greater routine availability and interpretability in epidemiological studies.

**Supplementary Table S5A. DLNM specifications for TEMP**

| **Number** | **df(time)** | **lag** | **cb(df)** | **degree** | **df(VISIB)** | **df(WDSP)** | **df(PRCP)** | **df(TD)** | **QAIC** | **QBIC** |
| --- | --- | --- | --- | --- | --- | --- | --- | --- | --- | --- |
| 1 | 12 | 3 | 2 | 2 | 2 | 3 | 2 | 3 | 258.2418 | 368.0617 |
| 2 | 12 | 3 | 2 | 2 | 2 | 2 | 2 | 3 | 258.8206 | 365.3126 |
| 3 | 12 | 3 | 2 | 2 | 3 | 3 | 2 | 3 | 260.3077 | 373.4555 |
| 4 | 12 | 3 | 2 | 2 | 3 | 2 | 2 | 3 | 260.8554 | 370.6753 |
| 5 | 12 | 3 | 2 | 2 | 2 | 3 | 2 | 2 | 262.8875 | 369.3795 |

**Supplementary Table S5B. DLNM specifications for DEWP**

| **Number** | **df(time)** | **lag** | **cb(df)** | **degree** | **df(VISIB)** | **df(WDSP)** | **df(PRCP)** | **df(TD)** | **QAIC** | **QBIC** |
| --- | --- | --- | --- | --- | --- | --- | --- | --- | --- | --- |
| 1 | 12 | 3 | 2 | 2 | 2 | 3 | 2 | 3 | 304.2697 | 414.0896 |
| 2 | 12 | 3 | 2 | 2 | 2 | 3 | 2 | 2 | 304.6556 | 411.1477 |
| 3 | 12 | 3 | 2 | 2 | 3 | 3 | 2 | 2 | 305.1439 | 414.9638 |
| 4 | 12 | 3 | 2 | 2 | 3 | 3 | 2 | 3 | 305.2742 | 418.4220 |
| 5 | 12 | 3 | 2 | 2 | 2 | 2 | 2 | 3 | 305.9484 | 412.4405 |

**Supplementary Table S5C. DLNM specifications for SLP**

| **Number** | **df(time)** | **lag** | **cb(df)** | **degree** | **df(VISIB)** | **df(WDSP)** | **df(PRCP)** | **df(TD)** | **QAIC** | **QBIC** |
| --- | --- | --- | --- | --- | --- | --- | --- | --- | --- | --- |
| 1 | 12 | 3 | 2 | 2 | 2 | 3 | 2 | 3 | 283.5949 | 393.4149 |
| 2 | 12 | 3 | 2 | 2 | 3 | 3 | 2 | 3 | 285.8839 | 399.0317 |
| 3 | 12 | 3 | 2 | 2 | 2 | 2 | 2 | 3 | 287.8799 | 394.3720 |
| 4 | 12 | 3 | 2 | 2 | 3 | 2 | 2 | 3 | 290.0947 | 399.9146 |
| 5 | 12 | 3 | 2 | 2 | 3 | 3 | 2 | 2 | 292.7870 | 402.6069 |

**Supplementary Table S5D. DLNM specifications for VISIB**

| **Number** | **df(time)** | **lag** | **cb(df)** | **degree** | **df(TEMP)** | **df(WDSP)** | **df(PRCP)** | **df(TD)** | **QAIC** | **QBIC** |
| --- | --- | --- | --- | --- | --- | --- | --- | --- | --- | --- |
| 1 | 12 | 3 | 2 | 2 | 2 | 3 | 2 | 3 | 248.1000 | 357.9199 |
| 2 | 12 | 3 | 2 | 2 | 2 | 3 | 2 | 2 | 250.1909 | 356.6830 |
| 3 | 12 | 3 | 3 | 2 | 2 | 3 | 2 | 3 | 251.3572 | 371.1608 |
| 4 | 12 | 3 | 3 | 2 | 2 | 3 | 2 | 2 | 251.5283 | 368.0039 |
| 5 | 12 | 3 | 3 | 2 | 3 | 3 | 2 | 3 | 251.6450 | 374.7764 |

**Supplementary Table S5E. DLNM specifications for WDSP**

| **Number** | **df(time)** | **lag** | **cb(df)** | **degree** | **df(TEMP)** | **df(VISIB)** | **df(PRCP)** | **df(TD)** | **QAIC** | **QBIC** |
| --- | --- | --- | --- | --- | --- | --- | --- | --- | --- | --- |
| 1 | 12 | 3 | 2 | 2 | 2 | 2 | 2 | 3 | 248.9175 | 355.4096 |
| 2 | 12 | 3 | 2 | 2 | 2 | 3 | 2 | 3 | 251.0876 | 360.9075 |
| 3 | 12 | 3 | 2 | 2 | 2 | 2 | 2 | 2 | 253.1265 | 356.2907 |
| 4 | 12 | 3 | 2 | 2 | 3 | 2 | 2 | 3 | 253.2095 | 363.0294 |
| 5 | 12 | 3 | 2 | 2 | 2 | 3 | 2 | 2 | 253.4442 | 359.9362 |

**Supplementary Table S5F. DLNM specifications for MXSPD**

| **Number** | **df(time)** | **lag** | **cb(df)** | **degree** | **df(TEMP)** | **df(VISIB)** | **df(PRCP)** | **df(TD)** | **QAIC** | **QBIC** |
| --- | --- | --- | --- | --- | --- | --- | --- | --- | --- | --- |
| 1 | 12 | 3 | 2 | 2 | 2 | 2 | 2 | 3 | 246.6851 | 353.1772 |
| 2 | 12 | 3 | 3 | 2 | 2 | 2 | 2 | 3 | 247.9119 | 364.3875 |
| 3 | 12 | 3 | 2 | 2 | 2 | 3 | 2 | 3 | 249.1986 | 359.0185 |
| 4 | 12 | 3 | 2 | 2 | 3 | 2 | 2 | 3 | 249.6473 | 359.4672 |
| 5 | 12 | 3 | 3 | 2 | 2 | 3 | 2 | 3 | 250.1805 | 369.9841 |

**Supplementary Table S5G. DLNM specifications for TD**

| **Number** | **df(time)** | **lag** | **cb(df)** | **degree** | **df(TEMP)** | **df(VISIB)** | **df(WDSP)** | **df(PRCP)** | **QAIC** | **QBIC** |
| --- | --- | --- | --- | --- | --- | --- | --- | --- | --- | --- |
| 1 | 12 | 3 | 3 | 2 | 2 | 2 | 3 | 2 | 279.3612 | 395.8369 |
| 2 | 12 | 3 | 3 | 2 | 2 | 2 | 2 | 2 | 280.7041 | 393.8519 |
| 3 | 12 | 3 | 3 | 2 | 2 | 3 | 3 | 2 | 282.0437 | 401.8472 |
| 4 | 12 | 3 | 3 | 2 | 2 | 3 | 2 | 2 | 283.3608 | 399.8365 |
| 5 | 12 | 3 | 2 | 2 | 2 | 2 | 3 | 2 | 285.3747 | 391.8667 |

**Supplementary Table S5H. DLNM specifications for PRCP**

| **Number** | **df(time)** | **lag** | **cb(df)** | **degree** | **df(TEMP)** | **df(VISIB)** | **df(WDSP)** | **df(PRCP)** | **QAIC** | **QBIC** |
| --- | --- | --- | --- | --- | --- | --- | --- | --- | --- | --- |
| 1 | 12 | 3 | 2 | 2 | 2 | 3 | 2 | 3 | 250.9974 | 360.8173 |
| 2 | 12 | 3 | 2 | 2 | 2 | 3 | 2 | 2 | 252.7601 | 359.2521 |
| 3 | 12 | 3 | 2 | 2 | 2 | 3 | 3 | 3 | 253.0143 | 366.1621 |
| 4 | 12 | 3 | 2 | 2 | 2 | 3 | 3 | 2 | 253.7628 | 363.5827 |
| 5 | 12 | 3 | 2 | 2 | 2 | 2 | 2 | 3 | 255.2827 | 361.7747 |

*Underlined values denote the optimal parameter settings selected for the final DLNM.

## 1.3 Forecasting Model Configuration

### 1.3.1 XGBoost Hyperparameter Settings

The XGBoost models were developed using a time-series forecasting framework. To avoid data leakage, the dataset was chronologically divided into training (70%), validation (10%), and testing (20%) subsets. Feature engineering included lag variables (1–5 weeks), moving averages (3- and 5-week windows), trend features, volatility features, and quantile-based risk indicators derived from meteorological variables.

Hyperparameters, including the number of boosting rounds (nrounds), maximum tree depth (max depth), learning rate (eta), minimum loss reduction (gamma), column subsampling ratio (colsample bytree), minimum child weight (min child weight), and row subsampling ratio (subsample), were optimized through grid search combined with time-series cross-validation. The final hyperparameter settings for each model are summarized in Supplementary Table S6.

Model performance was evaluated using a rolling one-step-ahead forecasting strategy, in which observed historical RSV positivity rates were used to construct lagged features for subsequent predictions.

**Supplementary Table S6. XGBoost Hyperparameter Settings**

| Model | nrounds | max depth | eta | gamma | colsample bytree | min child weight | subsample | features |
| --- | --- | --- | --- | --- | --- | --- | --- | --- |
| Base | 400 | 3 | 0.20 | 0.1 | 1 | 3 | 0.8 | 1 |
| Base_Enhanced | 400 | 4 | 0.01 | 0 | 0.6 | 1 | 0.8 | 14 |
| TEMP_Enhanced | 400 | 6 | 0.05 | 0 | 0.6 | 3 | 0.8 | 23 |
| DEWP_Enhanced | 400 | 4 | 0.01 | 0 | 0.6 | 3 | 0.8 | 23 |
| STP_Enhanced | 400 | 8 | 0.01 | 0 | 0.6 | 3 | 0.8 | 23 |
| SLP_Enhanced | 400 | 4 | 0.01 | 0 | 0.6 | 1 | 1 | 23 |
| VISIB_Enhanced | 400 | 4 | 0.05 | 0.1 | 0.6 | 3 | 0.8 | 23 |
| PRCP_Enhanced | 400 | 6 | 0.01 | 0 | 0.6 | 3 | 0.8 | 23 |
| WDSP_Enhanced | 400 | 8 | 0.05 | 0 | 0.6 | 3 | 1 | 23 |
| MXSPD_Enhanced | 400 | 6 | 0.01 | 0 | 0.6 | 3 | 1 | 23 |
| TD_Enhanced | 400 | 6 | 0.01 | 0 | 0.6 | 3 | 0.8 | 23 |

### 1.3.2 ARIMA Model Specifications

Stationarity of the RSV positivity time series was evaluated using the Augmented Dickey–Fuller (ADF) test. The optimal ARIMA model was identified using the auto.arima() function with maximum likelihood estimation, and the final model configuration is provided in Supplementary Table S7. Model performance was evaluated using a rolling one-step-ahead forecasting strategy, in which the model was re-fitted at each prediction step using all available historical observations and then used to predict the subsequent time point.

**Supplementary Table S7. ARIMA and SARIMA Model Specifications**

| Model | Order | ADF d |
| --- | --- | --- |
| ARIMA | (2,1,0) | 1 |

### 1.3.3 Revision of Model Performance Indicators

the Mean Absolute Scaled Error (MASE) was adopted as the main evaluation metric. MASE standardizes forecast errors relative to those produced by a benchmark forecasting method, thereby avoiding the instability of percentage-based metrics near zero values while maintaining straightforward interpretability. An MASE value below 1 indicates that the proposed model performs better than the corresponding benchmark forecast. In addition, MASE is scale-independent, allowing fair comparisons across regions, pathogens, and time series with different magnitudes. Therefore, MASE was additionally included as a model performance metric to provide a more robust evaluation of predictive accuracy.

#### 1.3.3.1 MASE_lag1

MASE_lag1 was calculated using a one-period lag naïve forecast as the benchmark model, where the observation at the previous time point serves as the prediction for the current time point. This metric evaluates the extent to which the proposed model improves upon short-term persistence forecasting.

#### 1.3.3.2 MASE_s52

MASE_s52 was calculated using a seasonal naïve forecast with a 52-week lag as the benchmark model. Given the pronounced annual seasonality observed in the weekly surveillance data, this metric assesses whether the proposed model outperforms forecasts based solely on recurrence patterns from the same period in the previous year.

# 2 Supplementary Figures

## 2.1 Correlation Analysis

**Supplementary Figure S1. Spearman Correlation Between RSV Positivity Rates and Meteorological Factors in Guangdong Province, 2019-2023**

## 2.2 Temporal Trends in Weekly RSV Activity

**Supplementary Figure S2. Temporal Trends in Weekly RSV Activity in Guangdong Province, 2019-2023**

(Average Positivity Rate = 12.72%)

## 2.3 Two-Dimensional Contour Visualization of Exposure–Lag–Response Associations

**Supplementary Figure S3. Non-linear Exposure–Response Relationships and Lag Effects of Meteorological Factors on RSV Transmission in Guangdong Province, 2019-2023**
